# 1842. Intravenous to Oral Antibiotic Stepdown for Uncomplicated Streptococcal Bacteremia

**DOI:** 10.1093/ofid/ofac492.1471

**Published:** 2022-12-15

**Authors:** Madison E Salam, Alison K Lew, Cynthia T Nguyen, Natasha N Pettit, Jennifer Pisano

**Affiliations:** University of Colorado Hospitals, Denver, Colorado; University of Chicago Medicine, Chicago, Illinois; University of Chicago Medicine, Chicago, Illinois; University of Chicago Medicine, Chicago, Illinois; University of Chicago Hospital, Chicago, Illinois

## Abstract

**Background:**

Historically, intravenous (IV) antibiotics have been the standard of care for the treatment of bloodstream infections (BSIs). IV antibiotic use predisposes patients to line-related complications and is associated with increased length of stay (LOS) and treatment costs as compared to oral (PO) antibiotics. The purpose of this study was to compare the efficacy and safety of IV therapy versus PO stepdown for uncomplicated Streptococcal BSIs.

**Methods:**

This single-center, retrospective, non-inferiority study included adults who had *Streptoccoccus* spp. in blood cultures with susceptibilities reported, < 48 hours of positive blood cultures, and were treated for the BSI. Patients were excluded if they had polymicrobial bacteremia, mortality prior to treatment completion, unattainable source control, or a complicated infection. The primary outcome was clinical success, defined as absence of infection recurrence, infection-related readmission, and infection-related mortality at 90 days. Secondary outcomes included individual components of composite endpoint, microbiological success, LOS, IV-line associated complications, and antibiotic-associated adverse events.

**Results:**

Of 79 patients included, 36 patients (45.6%) received IV for the full course of therapy and 43 (54.4%) received PO stepdown. Baseline characteristics were similar between groups. The most common sources were skin and soft tissue (34.6%) and oropharyngeal (13.6%). The median duration of IV therapy in PO stepdown patients was 4.4 days (IQR 2.7 – 6.9). Amoxicillin/clavulanate (44%) and cefdinir (24%) were the most common PO agents. Clinical success occurred in 94% of IV only patients and 91% of PO stepdown patients (p = 0.573). No infection-related deaths or recurrences occurred. Line complications were more frequent in patients receiving IV compared to PO stepdown therapy (14% vs. 0%, p = 0.015). LOS was lower in the PO stepdown group (11 vs. 6 days, p < 0.001).

**Conclusion:**

This study strengthens the hypothesis that PO stepdown therapy may be non-inferior to IV therapy for clinical success with a trend towards decreased LOS and line complications. However, this study was not powered to meet statistical significance. We are collaborating with several other centers to determine the true impact of this intervention.

**Disclosures:**

**All Authors**: No reported disclosures.

